# Supplementation with vitamin D improves the embryo quality in *in vitro* fertilization (IVF) programs, independently of the patients’ basal vitamin D status

**DOI:** 10.1007/s00404-024-07473-7

**Published:** 2024-04-05

**Authors:** Giorgio Maria Baldini, Michele Russo, Sara Proietti, Gianpiero Forte, Domenico Baldini, Giuseppe Trojano

**Affiliations:** 1 Momò Fertilife Clinic, Bisceglie, Italy; 2 R&D Department, Lo.Li. Pharma, 00156 Rome, Italy; 3 Department of Maternal and Child Health, Madonna Delle Grazie Hospital, 75100 Matera, Italy

**Keywords:** Vitamin D, ICSI, Embryo quality, IVF outcomes, Progesterone levels, Endometrial thickness

## Abstract

**Purpose:**

The study aims to demonstrate the effects of Vitamin D (VD) supplementation, prior to oocyte pick-up within IVF protocols, in women with diverse VD status at the enrollment.

**Methods:**

A total of 204 women eligible for intra-cytoplasmatic sperm injection (ICSI) cycles were included in the study and two homogeneous groups were selected from the database. Both group of patients with normal VD baseline level (> 40 ng/ml) and patients with low VD baseline level (< 20 ng/ml) were divided into control group and treatment group. The control group followed the standard procedure. The treatment group was supplemented with vitamin D3 as cholecalciferol in combination with Myo-Inositol, folic acid, and melatonin 3 months before standard procedure, once a day in the evening.

**Results:**

VD levels significantly increased in the study group of low baseline VD, both in serum and in the follicular fluid compared to controls. The treatment induced a significant improvement of the embryo quality in both group of patients considered.

**Conclusion:**

Supplementation of VD in patients undergoing ICSI procedures significantly improved the number of top-quality embryos compared with the control group, either starting from VD normal baseline values or starting from low values.

**Trial registration number:**

07/2018.

## What does this study add to the clinical work

**Table:** 

Women seeking pregnancy thorough IVF protocols, exhibit a significant improvement of embryo quality when Vitamin D is supplemented before starting the procedures, regardless of their baseline values of Vitamin D.	

## Introduction

Vitamin D (VD) incorporates a group of hormones with structural and functional similarities to progesterone [1]. Two of them are particularly relevant for the human body: *calcidiol* (or 25-hydroxyvitamin D_3_), which serves as circulating storage of VD; and *calcitriol* (or 1,25-dihydroxyvitamin D_3_), which is generally considered the hormonally active metabolite of VD [2], albeit all VD forms have a certain degree of affinity towards the VD receptor (VDR) [3]. Due to its relatively long half-life, *calcidiol* is routinely quantified within the blood to assess VD status; therefore, identifying whether a person has sufficient circulating levels of VD [4]. VD participates in physiological processes connected with female fertility and gestation [5, 6]. Studies on animal models, indeed, indicate that VDRs are expressed in the whole female reproductive system. In particular, VDRs are present in the cells of ovarian follicles [7] and in the endometrium [8], where VD supposedly cooperates with progesterone to regulate follicle physiological growth [1]. While still able to reproduce, VD-deficient rats and VDR-null mutant mice of reproductive age display reduced fertility and lower conception probability [9, 10].

Human granulosa cells also express VDR [11], and *in vitro* treatment with VD increases progesterone production and alters follicular sensitivity to anti-müllerian hormone (AMH), suggesting that VD may support the natural development of ovarian follicles [12]. Clinical investigations revealed that serum VD concentrations correlate with AMH levels, and that supplementation with VD helps counteracting AMH seasonal fluctuations in vivo [13]. Moreover, treating VD-deficient women with PCOS results in reduced levels of AMH in the serum, supporting the role of VD in the follicular physiology. *In vitro* data support this hypothesis, demonstrating that VD treatment restores normal processes in granulosa cells from women with PCOS [14].

Aside from its influence on follicular physiology, evidence has highlighted the role of VD from the periconception period and throughout human pregnancy. Indeed, VD enhances the receptivity of the endometrium towards embryos [15, 16], regulating endometrial growth and the decidualization of endometrial cells [17]. The activation of target genes directly involved in the implantation process, and the immunomodulatory activity of VD plays a crucial role for embryo implantation [18]. The same genes are also essential in the placentation process, suggesting that VD is one of the key regulators for physiological pregnancy. In fact, VD produced by the placenta targets VDRs in maternal decidua and in fetal trophoblasts, ensuring the crosstalk between the embryo and the endometrium in an autocrine and paracrine manner [19].

Whether VD status correlates with increased chances of achieving natural pregnancy is still unclear [20], this ambiguity may be due to the serum VD concentration thresholds commonly used as reference—i.e., < 20 ng/ml (deficiency); between 20 and 29.9 ng/ml (insufficiency); 30 ng/ml, or above (sufficiency). These thresholds are based on studies regarding bone health, and have been arbitrarily extended to all other applications, whereby they may not be suitable [21]. These studies point out a correlation between VD and ART outcomes, although causality cannot be inferred yet.

In contrast VD status appears to correlate with positive outcomes in assisted reproductive technology (ART). Indeed, serum VD levels reflect the concentrations in follicular fluid [22], representing a reliable marker for the ovarian VD reserve. Preliminary indications from observational studies, and further confirmations retrieved from robust meta-analysis found that the replete VD status of women undergoing *in vitro* fertilization (IVF) protocols is associated with increased biochemical and clinical pregnancy, and with an increased live birth rate [23–28]. Recent evidence indicates that an increase in serum VD, positively correlates with pregnancy outcomes in women over 36 years of age [29].

Interventional clinical trials that involve VD supplementation within IVF protocols highlighted better outcomes in the treatment group with respect to controls [30–34]. However, most of these studies were underpowered and presented several confounders, and thus should be considered primarily with further work required [18]. Interestingly, none of these studies investigated the effects of supplementation in women with sufficient serum VD levels. Indeed, information on potential harmful effects in these cases is unavailable, in addition to whether the currently adopted VD cutoffs are applicable to fertility outcomes.

This study aims to demonstrate the effects of VD supplementation, prior to oocyte pick-up within IVF protocols, in women with diverse VD status at the enrollment.

## Methods

### Study design and participants

This retrospective controlled study was carried out at Momò Fertilife center, in Bisceglie, Italy. Upon enrollment, all participants gave written informed consent. Data were stored and anonymized in a central database. Access to the study documentation system and the study database required user authentication and was restricted to the study staff only. The inclusion criteria were: age between 18 and 38 years; eligibility for Intra-cytoplasmatic sperm injection (ICSI) cycles. The exclusion criteria were: age over 38 years; BMI > 24.9; endocrine disorders interfering with metabolism of vitamin D; glucose intolerance; PCOS diagnosis; endometriosis; concomitant use of drugs altering the vitamin D metabolism; partner with severe oligospermia (less than 5 million/mL); partner with abnormal sperm morphology (> 96%); patients undergoing treatment during the summer months.

A total of 206 women eligible for ICSI cycles were included in the study and two homogeneous groups were selected from the database. Data analysis allowed us to stratify patients based on their basal levels of vitamin D. In detail, patients with normal VD baseline level (> 40 ng/ml) were divided as follow: control group (*N* = 52) and treatment group (*N* = 50); patients with low VD baseline level (< 20 ng/ml) were divided as follow: control group (*N* = 51) and treatment group (*N* = 53). The control group followed the standard procedure. The treatment group was supplemented with vitamin D3 as cholecalciferol (50 μg, 2000 IU) in combination with Myo-Inositol (600 mg), folic acid (200 μg), and melatonin (1 mg) (Inofolic® Luteal, Lo.Li. Pharma S.r.l., Rome, Italy) 3 months before standard procedure, once a day in the evening.

The primary endpoint defined for the study was the number of top quality embryo, while the secondary outcome were the level of VD in serum and Follicular fluids (FF), endometrial thickness, implantation rate, pregnancy rate, abortion rate.

We did not calculate the sample size of the study following the primary endpoint considering the poor clear evidence assessing this outcome, so we selected the patient corresponding to the inclusion criteria, trying to analyze homologous groups in terms of patient’s number.

### Ethics approval

The participants of the present study were all voluntarily enrolled and subscribed a written informed consent during their first clinical evaluation. The informed consent, described both the procedure’s risks and the complications potentially linked to metabolic abnormalities [35]. The study was regularly approved by the Local Ethical Committee of the Momò Fertilife Institute (approval number 07/2018) and conducted in accordance with the principles of the Declaration of Helsinki and Good Clinical Practice (GCP).

### Ovarian stimulation protocol and oocyte collection

Procedure previously described in Baldini et al. 2021 [29].

The eligible patients were treated with a controlled standard ovarian stimulation (COS) protocol using the recombinant follicle-stimulating hormone (rFSH) preparation (GONAL-f., Merck Serono, Darmstadt, Germany) in association with conventional gonadotropin-releasing hormone (GnRH) antagonist (Cetrotide, Merck Serono, Darmstadt, Germany) and human chorionic gonadotropin (hCG) (Gonasi, IBSA, Lugano, Switzerland) to induce ovulation.

### Serum and follicular fluids

Procedure previously described in Baldini et al. 2021 [29].

VD levels were measured at the beginning of treatment for each enrolled patient. Serum and FF were collected on the day of ovarian pick-up (OPU). FF was gathered with a 17G oocyte aspiration needle (Cook Medical, IN, USA) through a transvaginal ultrasound probe guide (VOLUSON S8, GE Healthcare, Boston, MA, USA), using a closed vacuum system tube commonly used for follicle aspiration. Following oocyte collection, obtained through a single ovarian puncture, exposure to a hyaluronidase solution (25 IU/ml) and the removal of the corona radiata were performed by repeated pipetting. The samples contaminated with blood traces were excluded from the analysis.

Metaphase II (MII) oocytes were selected using a stereomicroscope (Nikon SMZ 1500, Singapore), incubated in LGGF medium (Fertilization, Global, CooperSurgical, Trumbull, CT, USA), and injected with sperm (spermatic count eligible greater or equal to 5 million/mL) using an inverted microscope (Nikon Eclipse TE 200, Singaporean) at 400 ×  magnification. Subsequently, the fertilized oocytes were cultured in LGGG medium (Global, CooperSurgical, Trumbull, CT, USA) for 3–5 days until implantation. Luteal support was then provided after the transfer with intramuscular progesterone or vaginal micronized progesterone.

After oocyte recovery, the FF was collected into sterile tubes for a total amount of approximately 30 mL per patient, depending on the ovarian response to stimulation (max 14 follicles). Both plasma collected before follicle aspiration and FF were immediately delivered to the laboratory for VD measurement. Additionally, samples from plasma were also collected at baseline and handled as previously described.

### Vitamin D (VD) measurement

Procedure previously described in Baldini et al. 2021 [29].

Serum and FF from each patient were collected on the same day of the OPU, and then immediately sent to the laboratory for VD concentration evaluation using the VIDAS® 25OH VitD®. Total (BioMérieux SA, France). This test is a quantitative analysis that combines the ELISA method with final fluorescent detection (ELFA). Each determination of VD concentration was performed in triplicate.

The detection limit for VD concentration in both serum and FF was determined at 6.2 ng/ml. Intra-assay (Within-Run) *n* = 40; CV = 5.0%; Inter-assay (Run-to-Run) *n* = 10; CV = 7.8%. The linearity of the measurement range was defined from 7.1 to 126.2 ng/ml.

### Intra-cytoplasmic sperm injection (ICSI) and embryo transfer (ET)

Procedure previously described in Baldini et al. 2020 [35].

The ICSI procedure was performed at 37 °C under an inverted microscope (Nikon eclipse TE 200) at 400 ×  magnification. The ICSI was completed using an oil-hydraulic assisted microinjection system (Nikon eclipse TE 200). Embryo quality was evaluated according to Gardner scheme [36] that uses two letters (i.e. AA). The first letter indicates the Inner Mass quality (measuring the quality of expansion) with 4 grades: A, B, C, D. The second letter describe the trophoblast quality with the same 4 grades as for the first letter. Accordingly, top quality embryos were considered: AA, AB, BA, BB.

The ET procedure was performed after 3–5 days from OPU using a catheter (Guardia Access Embryo Transfer Catheter, Cook Medical) with transabdominal ultrasound guidance (General Electric, Logiq V2) for both transfer and implantation of one to two embryos.

No embryo was transferred on day 4 after OPU. We refer to common clinical practice suggesting the embryo transfer to be carried out on day 3 or 5 after OPU. The number of transferred embryos was assessed according to age, preferentially choosing a double transfer in patients with more than 38 years.

### Data analysis and statistics

The normal distribution of the data referred to each parameter measured in the study, was assessed using the Shapiro–Wilk Test. According to this analysis, the data did not follow a normal distribution.

Data recorded at baseline and after 3 months of treatment with Vitamin D in both datasets, were compared between the two groups considered for each dataset using the Mann–Whitney *U* test.

The implantation rate for each group of patients included in both databases was assessed with Chi-Square Test, while the abortion rate was evaluated using the Fisher’s Exact Test.

## Results

### Normal levels of Vitamin D

The baseline values of different parameters, including Antral Follicle Count (AFC), AMH, FSH, Luteinizing Hormone (LH), progesterone, and estradiol levels, were compared at baseline between the study and control group and no significant difference was reported. Results are shown in Table 1.

**Table 1:** Baseline values

	Control group	Study group
Age	35 (32–37)	34 (31–37)
BMI	22.6 (20.7–25.3)	22.3 (19.8–23.9)
AFC	12 (10–13.5)	12 (8–16)
AMH	1.8 (1.4–2.0)	1.9 (1.3–2.5)
FSH	7.3 (5.6–9.0)	6.5 (5.2–7.9)
LH	5.5 (3.9–7.8)	5.4 (3.7–7.5)
Estradiol	37.9 (32.5–52.8)	54 (28–60)
Progesterone	1 (0.7–1.3)	0.9 (0.7–1.5)
Vit. D	42 (32.2–48.2)	41.7 (34.9–51.4)

Table of baseline values of the group with normal VD baseline levels. Records indicated as median values, in parenthesis the values of 25th and 75th percentile

*BMI* body mass index, *AFC* antral follicle count, *AMH* anti-müllerian hormone, *FSH* follicle stimulating hormone, *LH* luteinizing hormone

The serum values of VD and estradiol were measured at baseline and after the treatment period (before oocyte pick-up) in both the control and study group, with no significant differences observed between the two groups. The VD status in the FF was also assessed, with no significant differences observed between the treated group and the controls.

Moreover, progesterone levels and endometrial thickness were measured before oocyte pick-up, exhibiting no significant differences between the treatment group and the control (Table 2).

**Table 2:** Comparison baseline vs. oocyte pick-up

	Control group		Study group	
	Baseline (T0)	Pick-up (T3)	Baseline (T0)	Pick-up (T3)
Estradiol	37.9 (32.5–52.8)	1308 (851–1780)	54 (28–60)	1479 (1052–2269)
Progesterone	1 (0.7–1.3)	1 (0.8–1.2)	0.9 (0.7–1.5)	0.9 (0.7–1.5)
Serum Vit. D	37.9 (32.5–52.8)	40.3 (32.3–46.1)	41.7 (34.9–51.4)	40.6 (33.2–50.9)
Follicular fluid Vit. D	–	39.7 (31.9–46.4)	–	41.4 (33.8–50.3)
Endometrial thickness	–	9.2 (8.6–10)	–	8.9 (8.3–10.4)

Effect of Vitamin D administration in women with normal VD baseline levels. Table of the values recorded at baseline (T0) and at oocyte pick-up after 3 months treatment with VD (T3) for both the study group and control group. Records measured at each timepoint indicated as median values, in parenthesis the values of 25th and 75th percentile

As it was among the outcomes of the ICSI procedures, the number of mature oocytes was recorded, with no significant difference observed between the two groups of patients considered. However, a significant difference was observed in the number of top-quality embryos, highlighting that treatment with VD before COS improves overall embryo quality. The findings from the present study did not reveal significant differences in the total number of embryos obtained and in the implantation rate. Furthermore, a non-significant reduction in the risk of abortion was observed following VD administration (Table 3).

**Table 3:** ICSI outcomes

	Control group	Study group
Num. mature oocyte	7 (5–8)	7 (6–8)
Num. top quality embryo	1 (0–2)	2 (1–3)*
Implantation rate	29.8%	28.6%
Abortion rate	35.2%	22.2%

Effect of Vitamin D administration on ICSI outcomes in women with normal VD baseline levels. Table of the values recorded at oocyte pick-up after 3 months treatment with VD (T3) for both the study group and control group. Records are indicated as median values, in parenthesis, the values of 25th and 75th percentile. Implantation rate and abortion rate are indicated as percentage related to each group of values considered

Statistical significance: *p < 0.05. *P* values are referred to the comparison of the study group vs. controls for the results recorded at each timepoint (Mann Whitney Test)

Taken together the data indicate that in these patients the VD administration did not lead to significant improvements in the observed parameters and the outcomes of ICSI procedures, with the exception of a significant improvement in embryo quality.

### Low levels of Vitamin D

The patients were compared for different parameters at baseline such as: AFC, AMH, FSH, LH, progesterone, and estradiol levels, and no significant differences were observed. Results are reported in Table 4.

**Table 4:** Baseline values

	Control group	Study group
Age	36 (34–38)	36 (34–39)
BMI	22.6 (20.9–25.3)	22.6 (21–25.4)
AFC	10 (8–11)	8 (6–13)
AMH	1.5 (1.2–1.8)	1.5 (1.1–2.0)
FSH	7.3 (5.6–9)	7.8 (5.7–10.3)
LH	5.5 (3.9–7.8)	5.4 (3.3–7.7)
Estradiol	37.9 (32.5–52.8)	48 (34.2–63)
Progesterone	0.8 (0.6–0.9)	0.7 (0.5–0.8)
Vit. D	17 (14.8–19.6)	16.9 (13.6–18.5)

Table of baseline values of the group with low VD baseline levels. Records indicated as median values, in parenthesis the values of 25th and 75th percentile

*BMI* body mass index, *AFC* antral follicle count, *AMH* anti-müllerian hormone, *FSH* follicle stimulating hormone, *LH* luteinizing hormone

The comparison of baseline VD levels in the two groups of patients enrolled for the study, highlighted no significant difference in the recorded values. In the study group, VD treatment for 3 months before oocyte pick-up, increased the level of serum VD, with a significant difference compared to the control. Indeed, we observe an increase in the study group, with a value of 40.4 ng/ml (34–44.6) reached after 3 months of VD administration. This was significantly different from the control group, which had a VD level with a value of 17.8 ng/ml (16–19.5) (Table 4). Furthermore, a significant difference was also observed in the VD levels measured in the FF. In the study group, the median VD level was 41.4 ng/ml (34.2–45.6), whereas in the control group, the median VD level was 17.8 ng/ml (15.9–19.8). Additionally, progesterone levels were significantly different between the two groups of patients, with a higher value of 0.9 ng/ml recorded in the study group (0.7–1.1) compared with 0.7 ng/ml in the controls (0.6–0.9). The measurement of endometrial thickness also revealed a significant difference between the patients enrolled for the study, with a median value of 9.4 mm after VD treatment (8.5–10.4), compared to a median value of 9 mm measured in the control group (8.4–9.4) (Table 5).

**Table 5:** Comparison baseline vs. oocyte pick-up

	Control group		Study group	
	Baseline (T0)	Pick-up (T3)	Baseline (T0)	Pick-up (T3)
Estradiol	37.9 (32.5–52.8)	1296 (851–1631.5)*	48 (34.2–63)	929 (666–1431)*
Progesterone	0.8 (0.6–0.9)	0.7 (0.6–0.9)t	0.7 (0.5–0.8)	0.9 (0.7–1.1)*
Serum Vit. D	17 (14.8–19.6)	17.8 (16–19.5)	16.9 (13.6–18.5)	40.4 (34–44.6)***
Follicular Fluid Vit. D	–	17.8 (15.9–19.8)	–	41.4 (34.2–45.6)***
Endometrial Thickness	–	9 (8.4–9.4)*	–	9.4 (8.5–10.4)*

Effect of Vitamin D administration in women with low VD baseline levels. Table of the values recorded at baseline (T0) and at oocyte pick-up after 3 months treatment with VD (T3) for both the study group and control group. Records measured at each timepoint indicated as median values, in parenthesis the values of 25th and 75th percentile

Statistical evaluation: * *p* < 0.05, *** *p* < 0.001. *P* values are referred to the comparison of the study group vs. controls for the results recorded at each timepoint (Mann Whitney Test)

The evaluation of the mature oocyte retrieved during the pick-up procedure, was not significantly different between the study group and the control group (Table 6).

**Table 6:** ICSI outcomes

	Control group	Study group
Num. mature oocyte	6 (5–8)	6 (5–9)
Num. top quality Embryo	1 (0–3)	2 (1–3)*
Implantation rate	35.3%	37.7%
Abortion rate	38.9%	20%

Effect of Vitamin D administration on ICSI outcomes in women with low VD baseline levels. Table of the values recorded at oocyte pick-up after 3 months treatment with VD (T3) for both the study group and control group. Records are indicated as median values, in parenthesis the values of 25th and 75th percentile. Implantation rate and abortion rate are indicated as percentage related to each group of values considered

Statistical significance: * *p* < 0.05. *P* values are referred to the comparison of the study group vs. controls for the results recorded at each timepoint (Mann Whitney Test)

Similar to the findings in the first dataset, VD administration significantly improved the embryo quality compared to controls. Indeed, we described a median value of 2 top-quality embryo for each patient (1–3), while the control group had a median value of 1 top-quality embryo for each patient (0–3).

A slight increase in the implantation rate was observed after VD treatment, even if the difference was not significant compared to the controls. The same trend was observed in the abortion rate, with a minor non-significant **decrease** in the percentage of abortion in the study group.

## Discussion

In this study, we aimed to address one of the most controversial issues in female infertility, which is whether VD supplementation may improve assisted reproduction outcomes, in particular overall embryo quality. It is well known that VD plays an important role in human physiology and pathology [37]. The presence of VDR in the uterus, endometrium, ovaries and placenta, evidence that VD has a unique role in the reproductive system [13]. Moreover, studies have demonstrated that VD deficiency is associated with female reproductive issues, including gestational diabetes, endometriosis, PCOS, infertility, and pregnancy complications [38–41]. It was also reported that VD levels could serve as a predictive marker for successful pregnancy following IVF, therefore its supplementation has become part of daily practice during assisted reproductive management, to improve ART outcomes [9, 42, 43]. However, despite extensive investigations over the past decades aimed at understanding the effects of Vitamin D supplementation in women experiencing infertility, no clear trend has been seen observed in prior results. The hypothesis that VD supplementation could enhance IVF results remains a subject requiring further exploration [44].

Patients in the described study were categorized into normal (> 40 ng/ml) and low-level groups (< 20 ng/ml) according to their baseline VD values. Results showed that VD supplementation, in patients with normal levels (> 40 ng/ml), significantly improved the number of top-quality embryos compared to the control group. Indeed, we observed an increase in median value of two top-quality embryo after VD administration versus one top-quality embryo in the controls (*p* < 0.05). While no statistically significant differences were noted in implantation rates, there was an interesting trend suggesting a reduction in the abortion rate for the study group. This is a surprising result compared to other studies in which VD supplementation did not yield significant benefits in terms of embryo quality [32, 45].

VD supplementation significantly improved overall embryo quality compared to the control group, regardless of baseline VD level. We observed a median value of two top-quality embryos after VD treatment, which was significantly higher than the one top-quality embryo retrieved in the control group (*p* < 0.05). Moreover, it is worth noting that, in patients with low baseline values, VD supplementation significantly increased the level of VD in the FF, with a median value of 41.4 ng/ml in the study group (34.2–45.6) with respect to a median value of 17.8 ng/ml in the control group (15.9–19.8) (*p* < 0.001). This finding highlights that peripheral VD status serves as an indicator of its availability within the ovary, and that serum and FF 25(OH)D are directly related to each other [46]. Patients with baseline low values of VD were also evaluated for serum VD levels, which exhibited a significant increase following supplementation. In the study group, the median serum VD level was 40.4 ng/ml (34–44.6) compared to a median value of 17.8 ng/ml in the control group (15.9–17.8) (*p* < 0.001).

These findings clearly demonstrate that supplementation with VD strongly enhances the bioavailability of the molecule, not only in the bloodstream but also in the ovary which is the specific target district investigated in this study.

Even if previous publications demonstrate the efficacy of VD supplementation in the improvement of IVF outcomes when administered with myo-Inositol, melatonin and folic acid [34, 47], our study was intended to highlight the benefit that might derive from VD especially in patients undergoing IVF who suffer of VD insufficiency.

In the low levels group, it is noteworthy that progesterone levels also significantly increased after VD supplementation compared to the control group. Interestingly, VD has been defined as a steroid hormone with progesterone-like activity [1]. Indeed, these molecules seem to support the implantation process and to sustain pregnancy through common signaling pathways, resulting in a synergistic activity which favors the gestational course. Particularly, the decidualization process requires elevated levels of estradiol and progesterone and the increase of ovarian steroid hormones, to support the cellular differentiation necessary for embryo implantation [48].

VD supplementation enhances progesterone release and increases the expression of progesterone receptors in a dose-dependent manner. This activity contributes to proper granulosa cell luteinization and sustained progesterone production, thus improving the endometrial status and facilitating decidualization [13]. Correct amount of progesterone also ameliorates IVF outcomes by sustaining critical processes as frozen ET [49].

VD supplementation may potentially increase endometrial receptivity. This effect is achieved by binding to its VDRs receptor on the endometrium, which in turn increases the expression of the HOXA10 gene, a crucial gene involved in both placentation and implantation [50, 51]. In a rat model, supplementation of VD positively influenced the proper endometrial priming required for successful implantation and the continuation of pregnancy by enhancing progesterone and HOXA 10 expression [52]. Indeed, after VD administration, an increase in HOXA-10 was observed together with an improvement in endometrial decidualization in a dose-dependent manner. This data indicates that VD and its receptor VDR directly modulate the expression of HOXA-10, thus supporting the implantation process and the achievement of a physiological pregnancy. Interestingly, HOXA-10 genes exhibit a dynamic temporal pattern of expression in adult female endometrium with an increased levels in the mid and late luteal phase, when the levels of estrogen and progesterone reach the highest values [53, 54].

Furthermore, in our study, supplementation with VD, investigated in patients with baseline low VD levels, resulted in a significantly higher value of endometrial thickness compared to the controls [median value: 9.4 mm (8.5–10.4) vs. median value: 9 mm (8.4–9.4); (*p* < 0.05)]. However, further investigation is required to determine whether the increase in endometrial thickness correlates with an increase in the implantation rate.

It has been highlighted that VD covers a key role in modulating the immune system during the decidualization process, which is fundamental for embryo implantation and development [19].

The expression of VDR on maternal decidua and fetal trophoblasts reflects an autocrine or paracrine activity of VD, which acts on both maternal and fetal sides, thus promoting embryo implantation [19]. VD not only mediates the correct tuning of immune defense to facilitate trophoblast invasion, but it also appears to be involved in providing essential nutrients, regulating hormone release, and eventually contributing to fetal growth and development [55].

The results from the present study highlight the potential positive role of VD, in facilitating the implantation process, which represents a critical step for the progression of a physiological pregnancy. The observed increase in both VD and progesterone levels following treatment seems to sustain the proper thickening of the endometrium, which in turn improves the micro-environment for embryo implantation. Interestingly, we observed a slight improvement in the implantation rate after VD administration in those women with low baseline VD levels, although this increase did not reach statistical significance within the study group. In addition, the abortion rate is not significantly different between the groups considered for the subset of women with low baseline VD levels. However, it is noteworthy that the abortion rate in the study group was 20% compared to a rate of 38.9% in the control group. The same trend was observed in the second subset of patients with normal baseline VD levels, where the abortion rate in the study group was 22.2% compared to 35.2% in the control group. While these data should be confirmed in a larger sample of patients, it is an interesting preliminary indication suggesting that VD may play a significant role in supporting patients seeking pregnancy and improving IVF outcomes.

In addition, an optimal sperm status and preparation is also necessary to maximize the chances of successful fertilization in IVF procedures [35, 56]. Interestingly, there are numerous studies showing that deficiency in VD not only affects sperm parameters but also affects sperm DNA integrity, which can subsequently affect the developmental competency of embryos [57]. In this context, certain limitations should be considered for the present study although we investigated VD status of women, VD concentration should also be assessed simultaneously in their male partner. Moreover, discrepancies within published works can be explained by various confounding factors, such as the source of VD (diet, exposure to the sun, former supplementation), lifestyle, ethnicity, age, BMI, seasonal variations, and the involvement of other ovarian factors. Additionally, some of the parameters analyzed in the datasets did not include both baseline and end-treatment records, thus preventing a comprehensive evaluation of all potential treatment influences on the selected parameters in the study. In addition, even if the greatest effects of the treatment observed in women with low baseline VD levels likely correlate with an effect due to VD supplementation, we cannot completely exclude that beneficial effect might be also related to the presence of myo-Inositol, melatonin and folic acid supplemented to the patients. In this regard, larger studies with control group treated with myo-Inositol, melatonin and folic acid are required to further confirm the results of the present study.

## Conclusion

The findings of this study revealed that supplementation of VD in patients undergoing ICSI procedures significantly improved the number of top-quality embryos compared with the control group, either starting from VD normal baseline values or starting from low values. In the latter case, VD supplementation also leads to improvements in other parameters such as progesterone levels, endometrial thickness, and VD levels in follicular fluid.

In summary, VD may provide valuable support for patients seeking pregnancy, since it improves various parameters when the baseline values are low and does not have detrimental effects when the baseline values are normal. On the contrary, the quality of embryos consistently improves following VD supplementation, regardless of the initial values.

## Data Availability

The data of this study are not publicly available due to privacy reasons but are available to the editors of the journal for review or query upon request.
