# Correction to: Supplementation with vitamin D improves the embryo quality in *in vitro* fertilization (IVF) programs, independently of the patients’ basal vitamin D status

**DOI:** 10.1007/s00404-024-07540-z

**Published:** 2024-05-03

**Authors:** Giorgio Maria Baldini, Michele Russo, Sara Proietti, Gianpiero Forte, Domenico Baldini, Giuseppe Trojano

**Affiliations:** 1 Momò Fertilife Clinic, Bisceglie, Italy; 2 R&D Department, Lo.Li. Pharma, 00156 Rome, Italy; 3 Department of Maternal and Child Health, Madonna Delle Grazie Hospital, 75100 Matera, Italy

**Correction to: Archives of Gynecology and Obstetrics** 10.1007/s00404-024-07473-7

In this article the authors name Giorgio Maria Baldini and Domenico Baldini were incorrectly written as Baldini Giorgio Maria and Baldini Domenico.

The original article has been corrected.

